# Physical Activity and Clinically Defined Arterial Hypertension in Consecutive Primary Care Patients: A Real-World Cross-Sectional Study

**DOI:** 10.3390/jcm15114049

**Published:** 2026-05-24

**Authors:** Peter M. Kalanin, Ivan Uher

**Affiliations:** 1 Department of General Medicine, Faculty of Medicine, Pavol Jozef Šafárik University in Košice, Rastislavova 45, 040 01 Košice, Slovakia; peter.kalanin@upjs.sk; 2 Institute of Physical Education and Sport, Pavol Jozef Šafárik University in Košice, 040 01 Košice, Slovakia; 3 MED-KAL, s.r.o., 040 01 Kosice, Slovakia

**Keywords:** arterial hypertension, cardiovascular prevention, clinically defined hypertension, cross-sectional study, physical activity, primary care, real-world data, self-regulation

## Abstract

**Background:** Arterial hypertension (AH) remains a leading modifiable risk factor for cardiovascular disease. Although the inverse association between physical activity (PA) and AH is well established, practice-based evidence from consecutive primary care populations remains clinically relevant for evaluating how this association appears under routine healthcare conditions. **Methods:** This retrospective cross-sectional study evaluated the association between self-reported PA and clinically defined AH in 1284 adult patients from routine primary care practice. PA was categorized according to World Health Organization recommendations as low (<150 min/week), moderate (150–300 min/week), or high (>300 min/week). AH was defined as a documented clinical diagnosis and/or ongoing antihypertensive treatment. Logistic regression was used to assess associations between PA category and AH, with adjustment for age, sex, body mass index (BMI), and LDL-C. **Results:** AH was present in 41.2% of the study population. AH prevalence differed significantly across PA categories, decreasing from 55.9% in the low PA group to 40.8% in the moderate PA group and 26.7% in the high PA group (*p* < 0.001). Compared with low PA, moderate and high PA were associated with lower odds of AH in crude analysis (OR = 0.54, 95% CI: 0.41–0.71; and OR = 0.29, 95% CI: 0.21–0.39, respectively). These associations remained significant after adjustment for age, sex, BMI, and LDL-C. **Conclusions:** Higher self-reported PA was associated with lower prevalence of clinically defined AH in consecutive primary care patients. The main contribution of this study is the replication and quantification of this established association in a real-world primary care cohort using pragmatic PA categories and routinely documented AH. Because of the cross-sectional design, these findings should be interpreted as associations and do not establish causality or directionality. Broader physiological and self-regulatory capacity may represent a hypothesis-generating direction for future research, but these processes were not directly measured in this study.

## 1. Introduction

Arterial hypertension (AH) remains one of the most prevalent and clinically important modifiable risk factors for cardiovascular disease worldwide, contributing substantially to morbidity, mortality, and healthcare burden [1,2,3,4]. Recent global estimates indicate that more than one billion individuals are affected by elevated blood pressure, with a substantial proportion remaining undiagnosed, untreated, or inadequately controlled [4,5]. Current European guidelines further emphasize the importance of early identification, classification, and management of elevated blood pressure and AH in clinical practice [6].

The pathophysiology of AH is complex and multifactorial, involving interactions between genetic predisposition, environmental influences, metabolic dysregulation, neurohormonal mechanisms, and autonomic regulation [7,8,9]. Recent evidence also suggests that subclinical cardiovascular dysfunction may be more pronounced in individuals with AH even in the absence of acute cardiovascular disease, further supporting the importance of early cardiovascular risk identification in routine clinical settings [10]. Among modifiable lifestyle factors, PA has been consistently identified as a cornerstone of cardiovascular prevention and health promotion [11,12,13,14]. A substantial body of evidence demonstrates that regular PA is associated with lower blood pressure, reduced cardiovascular risk, and decreased incidence of AH and cardiovascular disease [12,13,14].

Despite this well-established evidence, a substantial gap persists between recommended and actual PA levels in the general population. Global analyses indicate that many adults do not meet minimum PA recommendations, contributing to increased cardiovascular and metabolic disease burden [15]. This gap is particularly relevant in primary care, where patients frequently present with multiple comorbidities, variable adherence to lifestyle recommendations, and heterogeneous cardiovascular risk profiles.

Primary care represents a key setting for AH prevention, detection, and long-term management. As the first point of contact for most patients, primary care provides an opportunity to identify cardiovascular risk factors and reinforce lifestyle-related prevention strategies [16]. However, in routine clinical practice, PA is often less systematically assessed than pharmacological treatment or standard biomedical risk markers [17]. This is clinically relevant given persistent global concerns regarding insufficient PA levels and the need for practical intervention strategies [18]. Therefore, real-world evidence from primary care populations remains clinically relevant, particularly when PA is evaluated in relation to clinically documented AH rather than only continuous blood pressure measurements.

Although the inverse association between PA and AH has been extensively studied, the present study addresses a more pragmatic clinical question: how this established association appears in a consecutive real-world primary care population using routinely available clinical data and WHO-based PA categories. The added value of the present study does not lie in identifying a new PA–AH relationship, but in replicating and quantifying this association under routine primary care conditions using clinically defined AH as the outcome. This approach may support the practical value of PA assessment as part of cardiovascular risk evaluation in everyday clinical practice.

In addition to this empirical objective, sustained PA may also be considered from a cautious hypothesis-generating perspective. Chronic stress has been associated with increased allostatic load, impaired physiological regulation, and reduced capacity to sustain adaptive health behaviors over time [19,20,21]. Conversely, PA is known to influence several physiological systems relevant to cardiovascular regulation, including metabolic, vascular, autonomic, and inflammatory pathways [22,23]. However, stress physiology, autonomic regulation, allostatic load, and self-regulatory capacity were not directly measured in the present study. Therefore, any such interpretation remains conceptual and should not be considered an empirical conclusion of the current analysis.

Therefore, the aim of this study was to evaluate the association between self-reported PA and clinically defined AH in a consecutive real-world primary care population and to assess whether AH prevalence differed across PA categories. We hypothesized that higher PA levels would be associated with lower prevalence of AH after adjustment for key clinical variables.

## 2. Materials and Methods

### 2.1. Study Design and Setting

This study was designed as a retrospective cross-sectional analysis using routinely collected clinical data from a primary care setting. The real-world design reflects standard clinical practice and enables the evaluation of associations between PA and AH under naturalistic healthcare conditions without experimental manipulation.

The study was conducted in a general adult outpatient clinic providing comprehensive primary care services. The dataset represents consecutive patients attending routine visits, without preselection based on PA level or cardiovascular risk profile. This approach was used to better reflect a heterogeneous real-world primary care population [16]. Data were collected during the period from February 2021 to March 2026. The study was conducted in accordance with the Strengthening the Reporting of Observational Studies in Epidemiology (STROBE) guideline for observational research [24].

### 2.2. Study Population

A total of 1284 adult patients aged ≥18 years were included in the analysis. Patients were consecutively assessed during routine clinical visits within the defined study period. Eligible participants were required to meet the following inclusion criteria: age ≥ 18 years, available information regarding PA status, available information regarding AH status, and available clinical and laboratory variables required for multivariable analysis. Patients were excluded if they had incomplete clinical records, missing key variables, duplicate records, or acute medical conditions at the time of assessment.

### 2.3. Data Collection and Variables

Data were obtained from electronic medical records and collected as part of routine clinical assessment. All data were anonymized before analysis.

Demographic variables included age and sex. Clinical variables included BMI, LDL-C concentration, and AH status. BMI was calculated as weight in kilograms divided by height in meters squared (kg/m^2^). LDL-C values were obtained from standard biochemical laboratory analyses performed in certified laboratories. LDL-C measurements were extracted retrospectively from routine clinical laboratory records. Because this was a real-world retrospective analysis, detailed assay-kit and analyzer information was not systematically available for all patients. No study-specific commercial reagent, commercial cell lines, biological samples, or research-specific instruments were used in this study.

### 2.4. Definition of Arterial Hypertension

AH was defined based on a documented physician diagnosis in the medical record and/or ongoing antihypertensive treatment supported by available clinical documentation. Whenever available, documented diagnosis and blood pressure measurements were prioritized over medication use alone. Antihypertensive treatment was considered supportive rather than independently diagnostic in clinically uncertain situations. This pragmatic definition reflects real-world clinical decision-making and aligns with previous observational studies using routinely collected healthcare data [3,4].

### 2.5. Assessment of Physical Activity

PA was assessed using patient self-report as part of routine clinical evaluation. Patients reported their average weekly duration of moderate-to-vigorous PA. Based on World Health Organization recommendations, PA was categorized into three groups: low PA (<150 min/week), moderate PA (150–300 min/week), and high PA (>300 min/week) [25]. Although self-reported, this approach reflects routine clinical practice and has been widely used in epidemiological and primary care studies [12,15]. No standardized validated PA questionnaire was used, and detailed information on activity intensity, frequency, type, sedentary behavior, occupational activity, and recall period was not systematically available. Therefore, PA classification should be interpreted as a pragmatic self-reported clinical exposure rather than as an objectively measured behavioral variable.

### 2.6. Outcome Measures

The primary outcome was the presence of AH, coded as a binary variable (yes/no). Secondary analyses included descriptive assessment of AH prevalence across PA categories and the distribution of clinical variables within the study population.

### 2.7. Statistical Analysis

All statistical analyses were conducted using IBM SPSS Statistics, version 29 (IBM Corp., Armonk, NY, USA). Continuous variables are presented as means ± standard deviations (SDs), whereas categorical variables are presented as absolute frequencies and percentages. Normality of continuous variables was assessed using the Shapiro–Wilk test.

Differences in AH prevalence across PA categories were assessed using the chi-square (χ^2^) test. Differences in continuous clinical variables across PA categories were assessed using one-way analysis of variance (ANOVA).

To evaluate the association between PA and AH, binary logistic regression analysis was performed. PA categories were entered into the model with low PA as the reference category. Crude logistic regression analysis was first used to estimate unadjusted odds ratios (ORs). A multivariable logistic regression model was then used to assess whether PA remained associated with AH after adjustment for age, sex, BMI, and LDL-C. The multivariable model was constructed using clinically relevant covariates selected a priori rather than automated variable-selection procedures.

ORs with 95% confidence intervals (CIs) were calculated. Statistical significance was set at *p* < 0.05. Model assumptions were assessed by evaluating multicollinearity using variance inflation factors (VIF < 2), verifying linearity of continuous variables in the logit, and assessing model fit using the Hosmer–Lemeshow goodness-of-fit test.

### 2.8. Use of Artificial Intelligence-Assisted Tools

During manuscript preparation, ChatGPT based on GPT-5.5 Thinking (OpenAI, San Francisco, CA, USA) was used only for language editing, grammar checking, stylistic refinement, and formatting support. The tool was not used to generate, analyzed, or interpret study data. All scientific content, study design, statistical analyses, interpretation of results, and final manuscript content was critically reviewed and approved by the authors, who take full responsibility for the accuracy and integrity of the work.

### 2.9. Handling of Missing Data

The proportion of missing data was low (<5%) and was considered unlikely to substantially bias the findings. A complete-case analysis approach was applied.

## 3. Ethical Considerations

The study was based on retrospectively collected and fully anonymized clinical data. According to applicable Slovak legislation and institutional policies, formal ethical approval was not required for retrospective analysis of anonymized routine clinical data. The study was conducted in accordance with the principles of the Declaration of Helsinki (2013 revision).

## 4. Results

### 4.1. Baseline Characteristics of the Study Population

A total of 1284 adult patients were included in the analysis. The overall prevalence of AH in the study population was 41.2% (*n* = 529). Patients were stratified according to self-reported PA into three categories: low PA (*n* = 397), moderate PA (*n* = 497), and high PA (*n* = 390). Baseline demographic and clinical characteristics according to PA categories are presented in Table 1.

Significant differences across PA categories were observed for age, BMI, LDL-C, and AH prevalence. Patients reporting higher PA levels generally showed a more favorable clinical profile than those reporting low PA.

### 4.2. Hypertension Prevalence Across PA Categories

AH prevalence differed significantly across PA categories (Table 2). AH was most frequent in the low PA group, lower in the moderate PA group, and lowest in the high PA group. This represented a graded inverse cross-sectional association between PA category and AH prevalence.

### 4.3. Crude Association Between PA and AH

Crude logistic regression analysis was performed to quantify the unadjusted association between PA category and AH (Table 3). Low PA was used as the reference category. Compared with low PA, moderate PA and high PA were associated with lower odds of AH.

### 4.4. Adjusted Association Between PA and AH

Multivariable logistic regression analysis was performed to assess whether PA remained associated with AH after adjustment for age, sex, BMI, and LDL-C (Table 4). Low PA was used as the reference category. After adjustment, moderate and high PA remained associated with lower odds of AH. Age and BMI were positively associated with AH. Sex and LDL-C were not independently associated with AH in the adjusted model.

### 4.5. Model Diagnostics

Multicollinearity was not detected, with variance inflation factors below 2. Model calibration was assessed using the Hosmer-Leme show goodness-of-fit test. The adjusted model showed acceptable fit.

### 4.6. Summary of Key Findings

Overall, higher PA was associated with lower AH prevalence and lower odds of AH in a consecutive real-world primary care population. The association remained present after adjustment for age, sex, BMI, and LDL-C. Because of the cross-sectional design, these findings should be interpreted as associations and not as evidence of causality or directionality.

## 5. Discussion

### 5.1. Main Findings

The present study demonstrated a consistent inverse association between physical activity (PA) and arterial hypertension (AH) in a consecutive real-world primary care population. A graded cross-sectional association was observed, with AH prevalence decreasing from 55.9% among individuals with low PA to 40.8% among those with moderate PA and 26.7% among those with high PA. In multivariable analysis, both moderate and high PA remained associated with lower odds of AH after adjustment for age, sex, BMI, and LDL-C. The adjusted odds ratios were 0.58 for moderate PA and 0.33 for high PA compared with low PA.

These findings are consistent with established epidemiological evidence supporting the role of PA in cardiovascular prevention and blood pressure regulation [11,12,13,14,23,26]. The main contribution of the present study is not the identification of a new PA–AH relationship, which is already well established, but the replication and quantification of this association in a consecutive real-world primary care cohort using clinically defined AH and routinely available clinical variables.

Because the study was cross-sectional, the observed graded association should not be interpreted as evidence of causality or directionality. The findings indicate that higher self-reported PA was associated with lower AH prevalence under routine clinical conditions, but they do not establish whether higher PA reduced AH risk, whether individuals without AH were more able to maintain PA, or whether both processes contributed to the observed pattern.

### 5.2. Comparison with Existing Literature

The inverse association between PA and AH has been consistently demonstrated in prospective cohort studies, randomized trials, and meta-analyses [11,12,13,23]. Previous evidence has shown that regular PA is associated with lower blood pressure, improved cardiovascular outcomes, and reduced cardiovascular disease risk [13,23].

Recent large-scale and review evidence continues to support an inverse association between PA and cardiometabolic risk, including blood pressure-related outcomes [13,15,27,28,29]. Recent cross-sectional evidence by Luthy et al. also showed that higher Pa was associated with more favorable cardiovascular health metrics, including blood pressure-related components, in community-dwelling Black men [27]. Compared with that more detailed population-based analysis, the present study is narrower in scope but adds practice-based evidence from consecutive primary care patients with clinically documented AH. Thus, the present study does not claim to identify a new biological mechanism, but rather supports the clinical relevance of routine PA assessment in everyday primary care.

The findings are also consistent with the systematic review and meta-analysis by Wahid et al. [13], which quantified inverse associations between PA and cardiovascular disease and diabetes. While that review summarized broader cardiometabolic outcomes across multiple studies, the present analysis focuses specifically on clinically defined AH in a routine primary care setting. This provides practice-based evidence supporting the value of PA classification as part of cardiovascular risk assessment.

### 5.3. Real-World Primary Care Relevance

A major strength of this study is the use of routinely collected clinical data from a consecutive primary care population. Primary care settings include heterogeneous patients who are assessed and managed under everyday clinical conditions rather than under controlled research settings [15]. This makes the findings clinically relevant for routine cardiovascular risk evaluation.

The present study used pragmatic WHO-based PA categories and a clinically documented AH outcome. This approach reflects information that can be realistically obtained in primary care and may therefore be useful for routine risk stratification. The observed difference in AH prevalence between low and high PA categories suggests that PA assessment may help identify patients with different cardiovascular risk profiles in daily practice.

### 5.4. Hypothesis-Generating Regulatory Interpretation

PA is commonly conceptualized as a modifiable behavioral factor associated with cardiovascular health. The present findings are compatible with this interpretation. However, because of the cross-sectional design, PA may also be considered cautiously as a marker of broader physiological and self-regulatory capacity. Individuals who are healthier, less fatigued, or better able to sustain adaptive behaviors may be more likely to maintain regular PA over time.

Chronic stress and allostatic burden have been associated with impaired physiological regulation and reduced capacity to sustain health-promoting behaviors [19,20,21]. Conversely, PA is known to influence cardiovascular regulation through metabolic, vascular, autonomic, and inflammatory pathways [22,23]. However, stress physiology, autonomic regulation, allostatic load, and self-regulatory capacity were not directly measured in the present study. Therefore, this interpretation remains hypothesis-generating and should not be considered an empirical conclusion of the current analysis.

The graphical abstract illustrates this distinction by separating the observed cross-sectional association from the proposed regulatory interpretation, which requires direct testing in future longitudinal and interventional research.

### 5.5. Clinical and Public Health Implications

The findings support routine PA assessment in primary care as part of cardiovascular risk evaluation [17]. PA classification is simple, inexpensive, and clinically interpretable. Even when assessed by self-report, it may provide useful information about cardiovascular risk profiling in real-world practice.

However, because the study was observational and cross-sectional, the results should not be interpreted as evidence that increasing PA alone would necessarily reduce AH prevalence in this cohort. Longitudinal and interventional studies are needed to clarify whether changes in PA are followed by changes in AH risk under routine primary care conditions.

### 5.6. Limitations

Several limitations should be acknowledged. First, the cross-sectional design precludes causal inference, and reverse causation cannot be excluded. Therefore, it cannot be determined whether higher PA contributed to lower AH prevalence, whether individuals without AH were more able to maintain PA, or whether both processes operated simultaneously.

Second, PA was assessed using self-report as part of routine clinical evaluation. This may introduce recall bias, social desirability bias, overestimation of PA, and exposure misclassification. Detailed information on PA intensity, frequency, type of activity, sedentary behavior, occupational activity, and recall period was not systematically available.

Third, residual confounding cannot be excluded. The adjusted model included age, sex, BMI, and LDL-C, but several potentially relevant factors were not available or were not included in the present analysis, including antihypertensive medication class and adherence, smoking status, dietary patterns, alcohol intake, socioeconomic status, comorbid cardiovascular disease, renal disease, diabetes status, sleep quality, and psychosocial stress. Detailed assay-kit and analyzer information for LDL-C measurements was not systematically available because laboratory data were extracted retrospectively from routine clinical records. These unmeasured factors may have influenced both PA level and AH prevalence. Misclassification may also have occurred in patients using antihypertensive-class medications for indications other than AH, although documented clinical diagnosis and blood pressure measurements were prioritized whenever available. Detailed assay-kit and analyzer information for LDL-C measurements was not systematically available because laboratory data were extracted retrospectively from routine clinical records.

Fourth, the study was based on routinely collected real-world primary care data from a single outpatient setting, which may limit external generalizability. Finally, stress-related physiological processes, autonomic regulation, inflammatory markers, vascular biomarkers, allostatic load, and behavioral sustainability mechanisms were not directly assessed. Consequently, the proposed regulatory interpretation remains conceptual and hypothesis-generating rather than directly demonstrated by the data.

### 5.7. Future Directions

Future studies should use longitudinal and interventional designs to clarify the temporal relationship between PA and AH. A key question is whether higher PA contributes to better cardiovascular health over time, whether individuals with better health and lower physiological burden are more able to sustain PA, or whether both pathways operate bidirectionally. Objective PA measures, such as accelerometry or wearable-device data, would improve exposure assessment and reduce self-report bias.

Future research should also include more detailed information on PA intensity, frequency, type, sedentary behavior, and occupational activity. Studies integrating direct measures of stress physiology and autonomic regulation, such as heart rate variability and recovery dynamics, may help test whether regulatory capacity contributes to sustained PA behavior and cardiovascular outcomes [30]. Such studies could also evaluate whether self-reported PA functions as a pragmatic clinical indicator of broader regulatory capacity, allostatic load, and overall health status in primary care populations.

## 6. Conclusions

This retrospective cross-sectional study found that higher self-reported PA was associated with lower prevalence of clinically defined AH in a consecutive real-world primary care population. AH prevalence decreased across PA categories, with the highest prevalence observed in the low PA group and the lowest prevalence observed in the high PA group. These associations remained present after adjustment for age, sex, body mass index, and LDL-C.

The main contribution of this study is the replication and quantification of an established PA–AH association under routine primary care conditions using pragmatic PA categories and clinically documented AH. The findings support the routine assessment of PA as part of cardiovascular risk evaluation in primary care.

Because of the cross-sectional design, these findings should be interpreted as associations and do not establish causality or directionality. Broader physiological and self-regulatory capacity may represent a hypothesis-generating direction for future research on sustained PA behavior and cardiovascular health, but these processes were not directly measured or tested in the present study.

## Figures and Tables

**Table 1:** Baseline demographic and clinical characteristics according to physical activity categories.

Variable	Low PA (*n* = 397)	Moderate PA (*n* = 497)	High PA (*n* = 390)	*p*-Value
Age (years, mean ± SD)	58.4 ± 12.1	53.2 ± 11.4	48.7 ± 10.8	<0.001
Female sex, *n* (%)	207 (52.1)	261 (52.5)	194 (49.7)	0.412
BMI (kg/m^2^, mean ± SD)	31.2 ± 5.4	28.4 ± 4.8	25.9 ± 3.9	<0.001
LDL-C (mmol/L, mean ± SD)	3.42 ± 0.98	3.18 ± 0.91	2.97 ± 0.84	0.021
AH prevalence, *n* (%)	222 (55.9)	203 (40.8)	104 (26.7)	<0.001

Note: Values are presented as mean ± standard deviation or *n* (%), as appropriate. AH, arterial hypertension; BMI, body mass index; LDL-C, low-density lipoprotein cholesterol; PA, physical activity; SD, standard deviation.

**Table 2:** Arterial hypertension prevalence across physical activity categories.

PA Category	Total *n*	AH Yes, *n* (%)	AH No, *n* (%)
Low PA	397	222 (55.9)	175 (44.1)
Moderate PA	497	203 (40.8)	294 (59.2)
High PA	390	104 (26.7)	286 (73.3)

*p* < 0.001. Note: AH, arterial hypertension; PA, physical activity.

**Table 3:** Crude logistic regression analysis for AH according to PA categories.

Variable	OR	95% CI	*p*-Value
Low PA	Reference	—	—
Moderate PA vs. Low PA	0.54	0.41–0.71	<0.001
High PA vs. Low PA	0.29	0.21–0.39	<0.001

Note: AH, arterial hypertension; CI, confidence interval; OR, odds ratio; PA, physical activity.

**Table 4:** Adjusted logistic regression model for arterial hypertention.

Variable	Adjusted OR	95% CI	*p*-Value
Low PA	Reference	—	—
Moderate PA vs. Low PA	0.58	0.43–0.77	<0.001
High PA vs. Low PA	0.33	0.24–0.46	<0.001
Age	1.05	1.04–1.07	<0.001
Male sex	1.18	0.91–1.52	0.214
BMI	1.12	1.08–1.17	<0.001
LDL-C	1.08	0.97–1.21	0.110

Note: BMI, body mass index; CI, confidence interval; LDL-C, low-density lipoprotein cholesterol; OR, odds ratio; PA, physical activity.

## Data Availability

The datasets used and/or analyzed during the current study are available from the corresponding author upon reasonable request.
